# Fluoroless Rapid Mapping and Catheter Ablation of Intra-atrial Reentry Tachycardia in a Patient with Mustard Operation Using the Ensite™ Precision™ Electroanatomic Mapping System

**DOI:** 10.19102/icrm.2017.080901

**Published:** 2017-09-15

**Authors:** Bhavya Trivedi

**Affiliations:** ^1^Florida Hospital for Children, Orlando, FL

**Keywords:** Adult congenital heart disease, atrial arrhythmia, catheter ablation, fluoroless, 3D mapping

## Abstract

Catheter ablation of atrial arrhythmias in patients with atrial baffle palliation for dextro-transposition of the great arteries (requiring the Mustard or Senning procedures) can be challenging cases to complete, with long procedure times and high degrees of associated radiation exposure. Many ablation procedures can now be done using the fluoroless technique. The new EnSite™ Precision™ cardiac mapping system (Abbott Laboratories, Chicago, IL, USA) allows for the rapid mapping of arrhythmias and the performance of non-fluoroscopic procedures. This case report describes the use of this system in an adult patient undergoing Mustard operation, to rapidly map and successfully ablate intra-atrial reentry tachycardia with fluoroless technique.

## Introduction

Catheter ablation of atrial arrhythmias in patients with Mustard or Senning repair (for atrial baffle palliation for dextro-transposition of the great arteries (d-TGA)) can be challenging. The first published reports of catheter ablation in this population showed that patients can have multiple arrhythmia substrates, and most cases had long procedure and fluoroscopy times.^1–3^ To date, the incidence and recurrence after catheter ablation of atrial arrhythmias in adult Mustard and Senning patients remains high.^4–9^ Fluoroless catheter ablation of atrioventricular nodal reentrant tachycardia in a Mustard patient using three-dimensional (3D) electroanatomic mapping was recently reported.^10^ This current paper describes the first use of the EnSite™ Precision™ cardiac mapping system (Abbott Laboratories, Chicago, IL, USA) to allow for rapid mapping and to aid in fluoroless catheter ablation of intra-atrial reentry tachycardia (IART) in an adult patient with Mustard palliation.

## Case presentation

**Figure 1:**
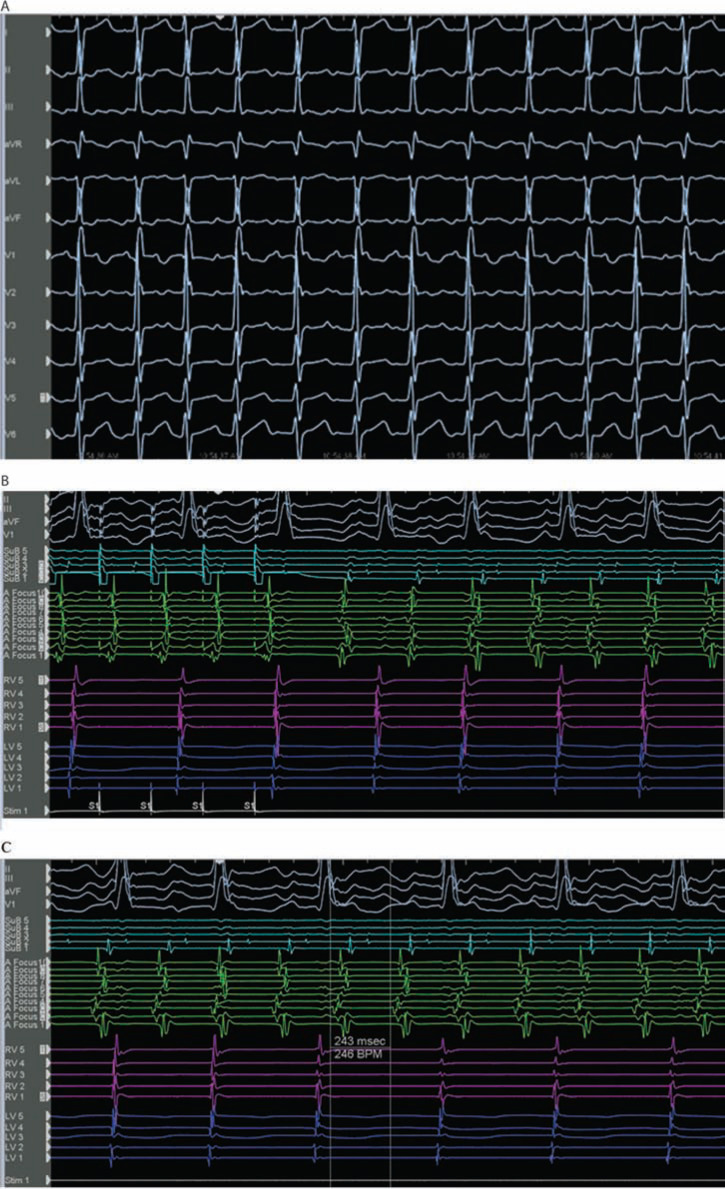
A 12-lead ECG and intracardiac electrograms with catheters in the right and left ventricles, superior baffle, and a duodecapolar mapping catheter in the anatomic left atrium. **A:** A 12-lead ECG of the IART. **B:** A demonstration of IART induction with atrial burst pacing from the superior baffle at 210 ms. **C:** IART with 2:1 atrioventricular conduction and a tachycardia CL of 243 ms.

**Figure 2:**
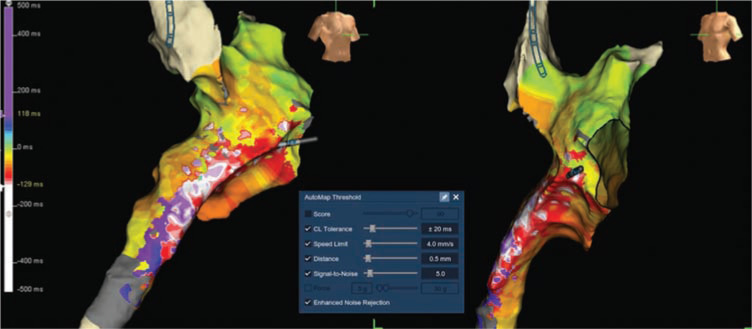
LAT map of IART with the EnSite™ Precision™ mapping system (Abbott Laboratories, Chicago, IL, USA). Note the “early” (white/red) mid-atrial diastolic region just behind the mitral annulus. This image also captures the automap settings (inset).

The patient in question was a 29-year-old male with d-TGA, status-post-Mustard procedure at the age of three years old. He had been on multiple antiarrhythmic medications over the last three years prior to the current case, and had never undergone an electrophysiology (EP) procedure before. He did have documented non-sustained wide-complex tachycardia on Holter monitoring and symptoms of palpitations over the previous three months, despite the administration of medical therapy. There was no clinical evidence of either sinoatrial node or atrioventricular node dysfunction, and a baseline 12-lead electrocardiogram (ECG) demonstrated sinus rhythm with incomplete right bundle branch block/right ventricular hypertrophy with strain pattern. A recent cardiac magnetic resonance imaging scan had revealed systemic right ventricle hypertrophy and mild dilation, with an ejection fraction of 48%. He was brought to the EP laboratory, and the procedure was completed under general anesthesia. IART was easily inducible with atrial burst pacing prior to the commencement of testing with isoproterenol **(Figure 1)**. The tachycardia was sustained, and activation mapping was immediately performed in tachycardia using the AutoMap™ module (Abbott Laboratories, Chicago, IL, USA). The tachycardia cycle length (TCL) was 243 ms **(Figure 1)**. Simultaneous local activation timing (LAT) mapping and model creation was performed using a 20 electrode Inquiry™ AFocus II™ catheter (Abbott Laboratories, Chicago, IL, USA), and then refined with the ablation catheter (8 mm Blazer^®^ catheter; Boston Scientific, Natick, MA, USA). A total of 3,264 LAT points and 2,530 voltage points were collected within three minutes, with minimal postprocessing required. The tachycardia circuit was immediately delineated and confirmed to be an IART/atrial flutter circuit, rather than a focal/microreentrant tachycardia **(Figure 2** and **Video 1)**. The LAT zero time was set at mid-atrial diastole with the roving activation interval defined to a range spanning 90% of the TCL. The propagation map revealed a timing gap where the activation circuit is coursing through the right atrium, which could not be sufficiently incorporated into the geometry with retrograde approach through the aortic and tricuspid valves. Nevertheless, the critical cavotricuspid isthmus portion of this right atrium (systemic side of the Mustard septation) was mapped, and it did not demonstrate mid-atrial diastolic or fractionated slowly propagating potentials.

**Figure 3:**
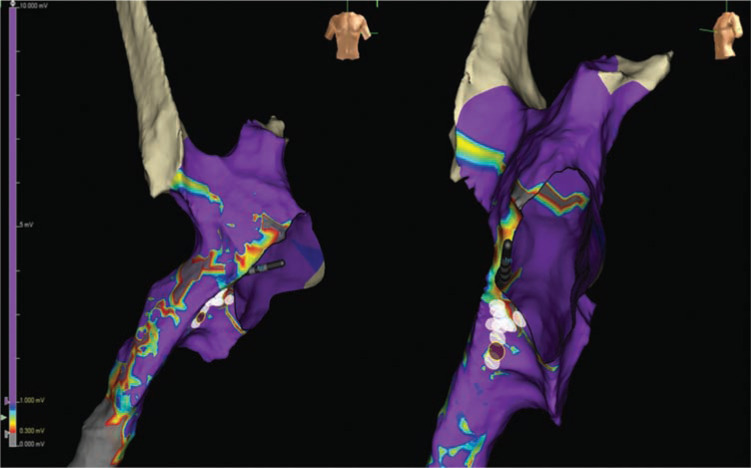
Voltage map during sinus rhythm demonstrating low-voltage “scar” regions, and showing the final line of ablation to terminate the arrhythmia.

**Figure 4:**
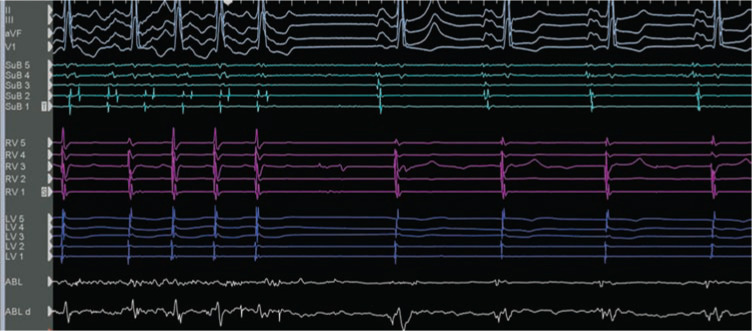
IART termination during the first RF lesion.

Voltage mapping was done during sinus rhythm while placing the initial catheters, and clearly demonstrated scar/low-voltage areas that could be anatomic substrates for IART **(Figure 3)**. In conjunction with the LAT propagation map, it was clear that the inferior area just behind the mitral valve was mid-atrial diastole and slowly propagating isthmic portion of the flutter circuit. Low-voltage/fractionated potentials in that location were 101 ms ahead of high left atrial (superior baffle) signals on the reference catheter. Surface ECG P-waves are typically low amplitude in these situations, but it was clear in this case that the signals in the presumed isthmus were mid-atrial diastolic. The first radiofrequency (RF) lesion just posterior to the mitral valve annulus terminated the arrhythmia within 47 s **(Figure 4)**, with a total lesion application time of 75 s. A short line of ablation was created from the anatomic mitral annular barrier to the posterior region of normal voltage to bisect the IART circuit **(Figure 3)**. The His-bundle location at the anteroseptal mitral annulus was identified with a quadripolar diagnostic EP catheter, and RF ablation was 8 mm to 10 mm inferior to that location (see His-bundle catheter in **Figure 3)**. This IART could not be reinduced in post-testing, even under the influence of isoproterenol. Though it is unusual for a macro-reentrant tachycardia to terminate with a single lesion, it is possible in this scenario that the initial RF lesion made with the 8-mm tip catheter may have eliminated the critical gap near the mitral annulus.

## Discussion

Several features of the new EnSite™ Precision™ cardiac mapping system (Abbott Laboratories, Chicago, IL, USA) were utilized in this case. The EnSite™ AutoMap module (Abbott Laboratories, Chicago, IL, USA) has parameter settings that aid in obtaining voltage and LAT rapidly and accurately, and in eliminating the need for post-collection manual refinement **(Figure 2, inset).** Surface ECG score threshold was not utilized with the low amplitude P-waves. The cycle length (CL) tolerance was set to ± 20 ms (from the template CL of 240 ms) to maintain CL matching. The speed limit tolerance was decreased to 4 mm/s, and was used to avoid erroneous collection during catheter collision. The distance threshold in this case was decreased to 0.5 mm so that points were collected only if the 3D position of the individual electrode moved a set distance or further from a previously collected point. Finally, the signal-to-noise ratio (SNR) threshold setting was set at 5.0 to limit the collection of mapping points to data in which the SNR is greater than 5.0. With these settings, the EnSite™ AutoMap module (Abbott Laboratories, Chicago, IL, USA) successfully collected over 3,000 LAT points within three minutes.

Rapid arrhythmia mapping helps to shorten the procedure time, and can be very useful to delineate arrhythmia circuits during hemodynamically unstable arrhythmias. For this patient, the total procedure time including anesthesia induction and emergence was 150 min. Antiarrhythmic medications were discontinued. At the one-week and the two-month follow-up, the patient reported no palpitations. Twenty-four-hour Holter monitoring two weeks post procedure showed no evidence of arrhythmia.

In conclusion, Mustard EP procedures and catheter ablations can be completed with the fluoroless technique. This case highlights the benefits of the use of the EnSite™ Precision™ mapping system (Abbott Laboratories, Chicago, IL, USA) for fluoroless catheter ablation of arrhythmias in adult congenital patients who have complicated anatomy and complex arrhythmia substrates.

## Figures and Tables

**Figure:**
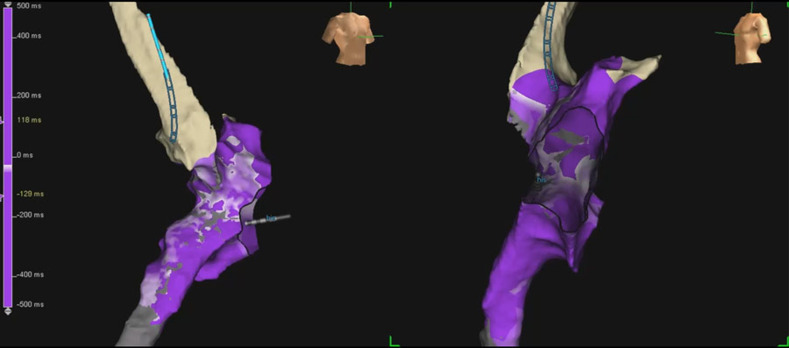
Play Video 1
